# Chemical Composition and Nematicidal Properties of Sixteen Essential Oils—A Review

**DOI:** 10.3390/plants10071368

**Published:** 2021-07-04

**Authors:** Trifone D’Addabbo, Pinarosa Avato

**Affiliations:** 1 Institute for Sustainable Plant Protection, National Council of Research, Via Giovanni Amendola 122/D, 70126 Bari, Italy; 2 Department of Pharmacy—Drug Sciences, University of Bari, Via Edoardo Orabona 4, 70125 Bari, Italy; pinarosa.avato@uniba.it

**Keywords:** aromatic plants, bionematicides, essential oils, fumigation, phytoparasitic nematodes, sustainable control, terpenes

## Abstract

Essential oils (EOs) can be a large source of new food-safe and healthy nematicidal products, due to their strong activity on crop pathogens and pests, including phytoparasitic nematodes, as well as to their low environmental persistence. This review summarizes the results from our 10-year studies on chemical features and nematicidal properties of 16 EOs with different botanical origins and compositions, i.e., the EOs from *Artemisia herba-alba* Asso (Asteraceae), *Cinnamomum camphora* (L.) J. Presl. and *Cinnamomum verum* J. Presl. (Lauraceae), *Citrus aurantium* L., *Cinnamomum. sinensis* L. Osbeck and *Ruta graveolens* L. (Rutaceae), *Eucalyptus citriodora* Hook, *Eucalyptus globulus* Labill. and *Syzygium aromaticum* (L.) Marry et Perry (Myrtaceae), *Mentha piperita* L., *Monarda didyma* L., *Monarda. fistulosa* L., *Rosmarinus officinalis* L. and *Thymus satureioides* Cosson (Lamiaceae), *Pelargonium asperum* Ehrh ex Willd (Geraniaceae) and *Schinus molle* L. (Anacardiaceae). All these EOs were chemically characterized and tested in vitro and/or in vivo for their activity against the phytoparasitic species *Meloidogyne incognita* Kofoid et White (Chitw.), *Pratylenchus vulnus* Allen et Jensen and *Xiphinema index* Thorne et Allen. Toxicity bioassays were conducted by exposing 2nd stage juveniles (*J2*) of *M. incognita*, mixed-age specimens of *P. vulnus* and adult females of *X. index* to 2–100 μg mL^−1^ concentrations of EOs or EO’s major constituents for 4–96 h and checking mortality effect after a further 24–72 h permanence in water. Egg hatchability bioassays consisted in exposing (24–48 h) *M. incognita* egg masses to 500–1000 mg mL^−1^ EO solutions followed by a 5-week hatching test in water. The in vivo experiments were undertaken in sandy soil strongly infested by *M. incognita* and treated with different doses of EOs, applied either in water solution or by fumigation. The effects of the treatments on nematode infestation on tomato and in soil were checked at the end of each experiment. Structure-activity relationships, as suggested by the different chemical compositions of tested EOs, were also highlighted. In agreement with literature data, our studies indicated that most of the tested EOs are highly suitable for the formulation of new safe nematicides, though still retarded by the lack of efficient stabilization processes and standardized EOs’ components and extraction techniques.

## 1. Introduction

Food safety and human health preservation require severe restrictions on the use of synthetic pesticides traditionally applied for the control of crop pathogens and pests, due to their hazardous effects on soil, animals, and humans [1,2]. Phytochemicals from a large variety of plants have received an increasing interest as an alternative strategy for phytoparasitic nematode management [3,4,5,6]. Within plant-derived nematicidal compounds, a major role, as both a research topic and a source of new nematicides, has been increasingly provided to essential oils (EOs) from a wide range of aromatic and medicinal plants [7,8,9]. EOs’ activity has been extensively documented on root-knot nematodes of the genus *Meloidogyne*, as the phytonematode species economically most damaging and prone to worldwide spread [10,11,12], as well as on the pinewood nematode *Bursaphelenchus xylophilus* (Steiner and Buhrer) Nickle, due to the serious phytosanitary problems raised by this nematode in the pine forests of South Korea and Portugal [13,14,15]. Adversely, a minor attention was provided to EOs’ activity on other potentially harmful phytonematodes, such as the cyst-forming (*Heterodera* spp. and *Globodera* spp.) and root-lesion (*Pratylenchus* spp.) species or the stem nematode *Ditylenchus dipsaci* (Kühn) Filipjev [16,17,18,19]. The 10-year studies of our research group were addressed to characterize the chemical features and nematicidal properties of 16 EOs from different botanical and geographic origins, as well as their structure–activity relationship. Main findings from these studies are reviewed and commented in this work in comparison with the related literature data.

## 2. Essential Oils

EOs are mixtures of volatile lipophilic constituents generally produced by the specialized metabolism of aromatic and medicinal plants from a wide range of botanical families (Lamiaceae, Myrtaceae, Rutaceae, Apiaceae, and others) as responsible for their distinctive odor, flavor or scent, though also present in non-vascular plants such as some liverworts [20]. EOs are stored in plant secretory epithelial or parenchimal cells, forming structures of various kinds such as glandular trichomes or excretory idioblasts. The ecological role of EOs is still not clearly defined, though plant secretory products have been suggested to provide adaptive benefits, including plant protection against phytopathogens and parasites [21].

In general, EOs’ constituents are made up of relatively inert chemicals, consisting mainly of carbon and hydrogen, with one or more functional groups that provide alcohols, aldehydes, ketones, esters, and other chemical types contributing at different concentrations to an entire EO’s composition, with some of them present in very high amounts (up to 80%) and others only as traces.

Terpenoids are the most important group of specialized components of plant EOs and are principally represented by mono- and sesquiterpene compounds, sometimes associated with low molecular weight phenylpropanoids [22]. Plant terpenoids are synthesized via two different metabolic pathways: the mevalonate (MVA) pathway in cytosol leads to the formation of sesquiterpenoids, while the methylerythritol phosphate (MEP) pathway occurring in chloroplasts results in the synthesis of monoterpenoids. Phenylpropanoids derive instead from the shikimate pathway mainly occurring in chloroplasts [23].

**Figure 1:**
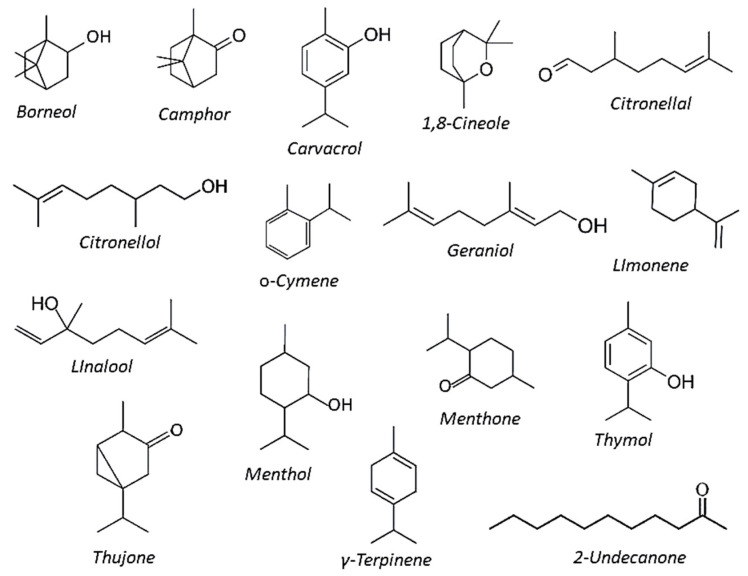
Chemical structures of selected EOs’ components.

Plant EOs can be also characterized by phytochemical polymorphism, as individual plants from the same species can produce several chemotypes with different EOs’ compositional profiles, possibly resulting from diversification of EOs biosynthetic pathways under different environmental conditions [20]. This phenomenon was clearly evidenced within the Lamiaceae plants, in which several chemical types were identified in *Origanum*, *Lippia*, *Mentha*, *Lavandula*, and *Ocimum* species, though chemotypes were detected also within the Asteraceae, such as among *Matricaria*, *Tagetes*, and *Achillea* species. A summary of some of the most common components in plant essential oils is depicted in Figure 1.

Biological and pharmacological activity of EOs are often related to their main constituents, though minor components may also play a relevant role and sometimes act in a synergistic or antagonistic way with major components. Moreover, EOs’ biological and pharmacological effects are also related to their lipophilicity, which allows them to easily enter cells and interfere with membranes’ structure and fluidity by an increased permeabilization.

Products investigated in our studies were both commercial pure EOs from *Eucalyptus citriodora* Hook, *E. globulus* Labill. *Citrus aurantium* L., and *Ruta graveolens* L. (Rutaceae), *Mentha piperita* L. (Lamiaceae), *Pelargonium asperum* Ehrh ex Willd (Geraniaceae)*, Cinnamomum camphora* (L.) J. Presl. and *C. verum* J. Presl. (Lauraceae), *Schinus molle* L. (Anacardiaceae), and *Syzygium aromaticum* (L.) Marry et Perry (Myrtaceae), as well as EOs directly extracted by hydrodistillation from wild Moroccan plants of *Artemisia herba-alba* Asso (Asteraceae), *Citrus sinensis* L. Osbeck (Rutaceae), *Rosmarinus officinalis* L. and *Thymus satureioides* Cosson (Lamiaceae) or cultivated plants of two *Monarda* species, i.e., *M*. *didyma* L. and *M. fistulosa* L. (Lamiaceae) [24,25,26,27].

The GC and GC-MS analysis of these EOs highlighted largely differentiated compositional profiles, mainly consisting of high concentrations of oxygenated monoterpenes (Figure 1). Carvacrol was present in the EOs from *M. dydima*, *M. fistulosa*, and *R. graveolens* (14, 24 and 15%, respectively), whereas its isomer thymol was among the monoterpene components of EOs from *T. satureidoides, M. dydima*, and *M. fistulosa* (12%, 6%, and 8%, respectively). However, EOs from *M. didyma* and *M. fistula* were also characterized by relevant amounts of γ-terpinene (22% and 25%, respectively). Large amounts of 1,8-cineole (*syn* eucalyptol) were found in the EOs of *R. officinalis* and *E. globulus* (47% and 92%, respectively) and in the EO of *C. camphora* (22%). Camphor was detected in the EOs of *A. herba-alba* (26%) and *R. officinalis* (12%), whereas main constituents of *P. asperum* EO were citronellol (35%) geraniol (22%) and linalool (13%).

The thujone isomers *cis*-thujone and *trans*-thujone were detected only in the *A. herba-alba* EO (25% and 16%, respectively), as well as borneol was present (29%) only in *T. satureidoides* EO, *o*-cymene only in *M. didyma* and *M*. *fistulosa* EOs (13% and 11%, respectively), and menthol, menthone, and isomenthone only in the EO from *M. piperita* (55, 20 and 11%, respectively). The hydrocarbon monoterpene limonene was almost the unique constituent of *C. aurantium* (95%) and *C. sinensis* (96%) EOs, though largely abundant (59%) also in *C. camphora* EO.

EOs of *S. aromaticum* and *R. graveolens* were prevalently constituted of eugenol (90%) and the aliphatic ketone *2*-undecanone (83%)*,* respectively. Analogously, citronellal prevailed in *E. citriodora* EO (84%) and the phenylpropanoid *E*-cynnamaldehyde (85%) in the EO of *C. verum*, which also included 13% of eugenol (Figure 1). In contrast, the EO of *S. molle* consisted of three main components, i.e., α-pinene, linalool, and eugenol, present at almost equal amounts: (15%, 10% and 12%, respectively).

## 3. Phytoparasitic Nematodes

Plant-parasitic nematodes are among the most serious constraints to world agriculture, globally causing damages estimated at USD 80 billion per year, most of which is due to root-knot species of the genus *Meloidogyne* [28]. Moreover, these losses are presumably underestimated due to nonspecific symptoms and difficult recognition of nematode attacks [29].

**Figure 2:**
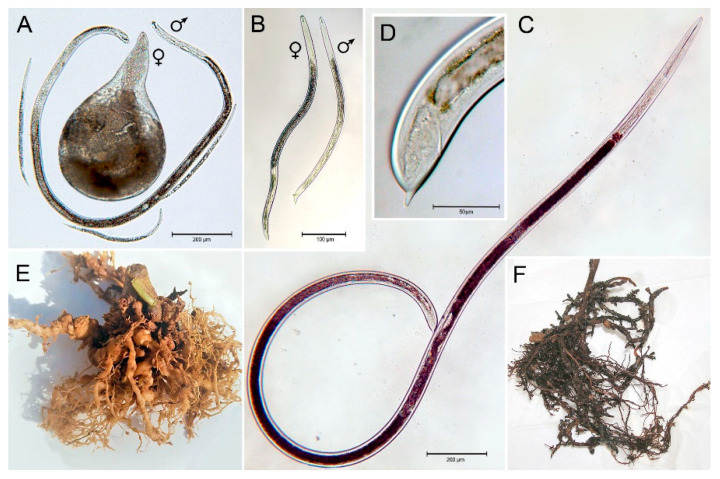
Morphology and symptoms of the investigated nematode species: (**A**) juvenile, male and female of *Meloidogyne incognita*; (**B**) male and female of *Pratylenchus vulnus*; (**C**,**D**) whole body and tail of *Xiphinema index*; (**E**) tomato roots infested by *M. incognita*; (**F**) grapevine roots infested by *X. index*. Courtesy of Dr. Alberto Troccoli, IPSP-CNR, Bari, Italy.

Among the about 98 root-knot nematodes species included in the genus *Meloidogyne*, *M. incognita* Kofoid et White (Chitw.) is unanimously considered the most economically harmful as it is highly destructive on a wide range of herbaceous and tree crops [30] (Figure 2).

A worldwide distribution on a large number of host crops is also presented by root lesion nematodes of genus *Pratylenchus*, quite rightly included among the most devastating nematode pests. In particular, *P. vulnus* Allen et Jensen is a severe parasite of fruit trees widespread in commercial orchards and nurseries of the Mediterranean region and United States [31].

The dagger nematode *Xiphinema index* Thorne et Allen is an ectoparasite species distributed throughout the world which feeds on grapevine root tips and directly causes root swelling and gall formation with a consequent reduction of plant growth. However, the economic impact of this species is mainly related to its vehiculation of dangerous grapevine viruses, such as the grapevine fanleaf virus [32].

In our studies, in vitro toxicity bioassays were conducted on 2nd stage juveniles (*J2*) of *M. incognita*, mixed-age specimens of *P. vulnus* and *X. index* females, which were exposed for 4–96 h intervals to 2–100 μg mL^−1^ concentrations of EOs or their single constituents [26,27]. Egg hatchability bioassays were undertaken on *M. incognita* egg masses treated for 24–48 h with 500–1000 μg mL^−1^ EO solutions [27]. Finally, in vivo studies on tomato (*Solanum lycopersicum* L.) were carried out in soil infested by *M. incognita* (20 eggs and *J2* mL^−1^ soil) and treated with 50–200 μg kg soil^−1^ doses of the different EOs, applied either in water solution or by fumigation [24,25,26,27].

## 4. Nematicidal Activity of Experimental EOs

**Figure 3:**
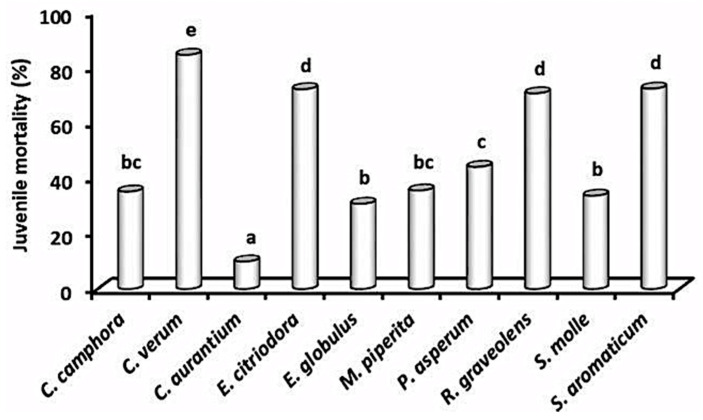
Aggregated mortality of *M. incognita* juveniles after a 24 h treatment with 0. 78–100 μg mL^−1^ solutions of 10 different essential oils.

The EOs from *A. herba-alba* and *R. officinalis* were highly active on *M. incognita J2* and *X. index* females, as both resulted in an almost complete mortality after a 96 h exposure to only 2 μg mL^−1^ solutions, but were less toxic to *P. vulnus* [25]. The lesion nematode *P. vulnus* was less sensitive than *M. incognita* and *X. index* also to the EO from *T. saturejoides* and both *Monarda* EOs [25,26]. The two *Cinnamomum* EOs were differently active on *M. incognita J2*, as a similar 64% mortality occurred after a 24 h *J2* exposure to 0.78 and 25 μg mL^−1^ solutions of *C. verum* and *C. camphora*, respectively [27] (Figure 3).

Analogously, toxicity to *M. incognita J2* largely differed between the two *Eucalyptus* EOs, as only an 8 h exposure to a 12.5 μg mL^−1^ solution of *E. citriodora* EO was enough to cause more than 90% *J2* mortality, while similar rates were reached only after a 24 h immersion in a 100 μg mL^−1^ solution of the *E. globulus* EO.

A moderate toxicity to root-knot nematode *J2* was recorded for the EOs of *M. piperita*, *S. molle*, and *P. asperum*. Results showed more than 80% mortalities only at concentrations ≥50 μg mL^−1^, whereas a strong activity was provided by the EOs of *R. graveolens* (90% *J2* mortality after a 8 h exposure to a 12 μg mL^−1^ solution) and *S. aromaticum* (30% mortality after a 24 h permanence in ≤6.25 μg mL^−1^ EO solutions) [27].

Both *C. sinensis* and *C. aurantium* EOs were weakly active on *M. incognita*, while *C. sinensis* EO showed a consistently higher toxicity to *P. vulnus* (more than 73% peak mortality) [25,27]. Adversely, solutions of both *Monarda* EOs were strongly toxic to *M. incognita J2* but less active on *P. vulnus*, reaching 80–83% mortality rates after 24 h exposures to 12.5 and 100 μg mL^−1^ concentrations, respectively [26]. The 24 h LD_50_ values indicated EOs of *A. herba-alba* and *C. verum* as the most toxic to *M. incognita J2*, followed by the two *Monarda* EOs and EOs from *E. citriodora, R. graveolens,* and *S. aromaticum*, while the poorest activity was confirmed for the EOs from the two *Citrus* species (Table 1).

**Figure 4:**
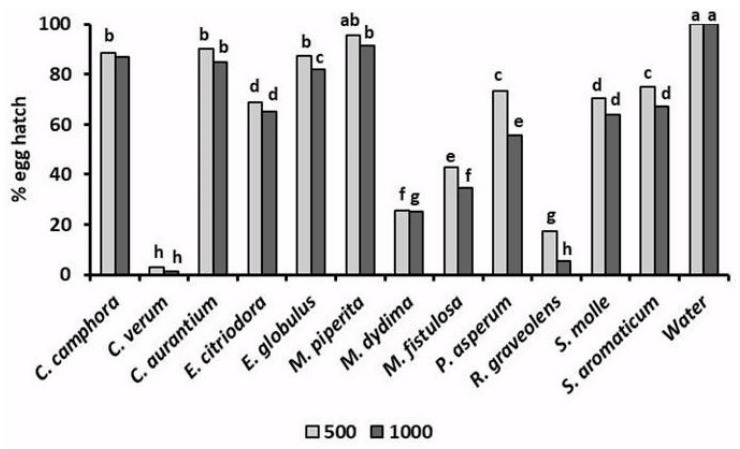
Relative hatchability of *M. incognita* eggs after a 48 h egg mass exposure to 500 or 1000 μg mL^−1^ solutions of 12 different essential oils.

In addition to their toxicity to infective *J2*, the tested EOs also variously affected the hatchability (Figure 4) of root-knot nematode eggs [26,27]. A 96 h exposure of *M. incognita* egg masses to a 500 μg mL^−1^ solution of EOs of *C. verum* and *R. graveolens* reduced the percentage of egg hatch to only 1.2% and 7.0%, respectively. Analogously, egg hatchability was strongly limited by similar treatments with *M. didyma* and, at less instance, *M. fistulosa* EOs. A lower but significant egg hatch inhibition was also caused by the two *Eucalyptus* EOs, as well as by the EOs of *P. asperum*, *S. molle*, and *S. aromaticum*. Adversely, poor or nil effects on root-knot nematode egg viability were found for *C. aurantium* and *M. piperita* EOs.

**Figure 5:**
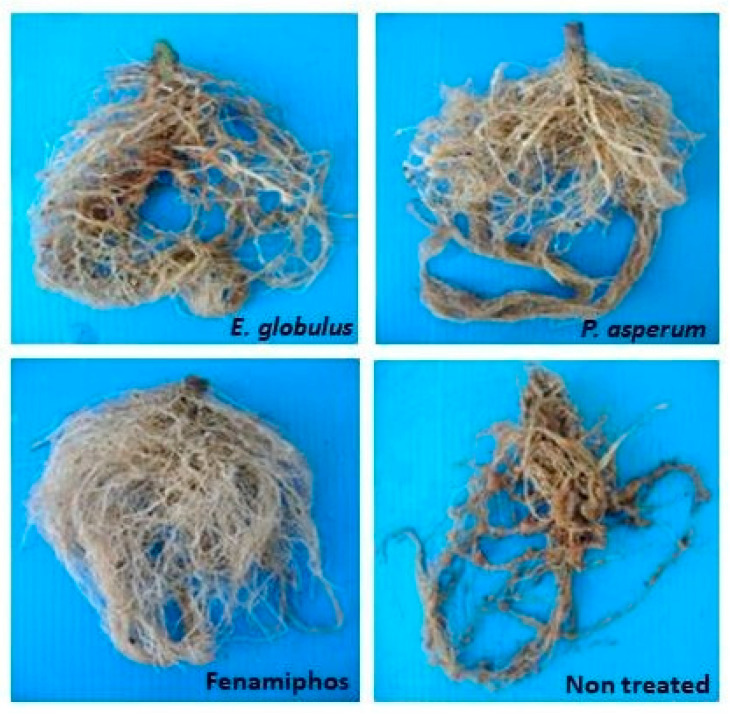
Tomato roots from soil infested by *M. incognita*, non-treated and treated with EOs from *E. globulus* and *P. asperum* (100 μg Kg^−1^ soil), or nematicide fenamiphos.

**Figure 6:**
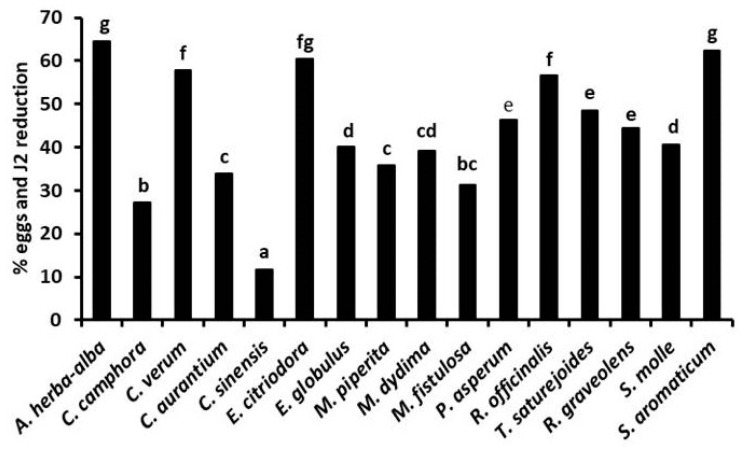
Relative suppression (non-treated soil = 0) of *M. incognita* multiplication on tomato roots after soil treatment with a 100 μg Kg^−1^ soil dose of 16 different essential oils.

In the experiments in soil, the strongest suppression of gall formation and nematode egg density on tomato roots (Figure 5) occurred after soil treatments with the EOs from *E. citriodora*, *E*. *globulus, M. piperita*, *P*. *asperum,* and *R. graveolens*, applied at 50–200 μg kg^−1^ soil doses either by fumigation or in an aqueous suspension [24,27]. Nematode infestation on tomato roots was also strongly suppressed after soil fumigation with the same range of doses of *A. herba-alba*, *R. officinalis*, and *T. satureioides* EOs [25], as well as by soil irrigation with 50–200 μg kg^−1^ soil doses of EOs from *S. aromaticum*, *C. verum*, and *E. citriodora* or *M. didyma* and *M. fistulosa* EOs [26,27] (Figure 6). Adversely, the lowest suppressive performance was provided by the soil treatments with *C. aurantium* and *C. sinensis* EOs, in good agreement with the poor or limited in vitro activity of these two EOs.

No literature data were available on the nematicidal activity of *C. aurantium*, *S. molle,* and *Monarda* EOs, while only single in vitro assays stated a limited toxicity to root-knot nematode *J2* and eggs of EOs from *C. sinensis* and *A. herba-alba* and, adversely, a strong toxicity of *T. satureioidoes* EO [33,34].

Previous reports on the nematoxicity of the other EOs investigated in our studies were mostly referred to in vitro assays on root-knot nematode species with EO samples not always chemically characterized, while toxicity to other phytonematode species was poorly or not at all documented. Most of these studies generally agreed with data from our experiments.

EOs from *Eucalyptus* species, also including *E. citriodora* and *E. globulus*, were always found to reduce *J2* motility and viability and egg hatchability of different *Meloidogyne* species [12,35,36]. In agreement with our data, nematicidal properties of *Cinnamomum* EOs were described as consistently variable among the species, with a moderate suppressiveness of *C. camphora* EO to *M. incognita* on tomato and a strong in vitro toxicity of the EO of *C. verum* to the root-knot species *M. incognita* and *M. graminicola* Golden and Birchfield and the pinewood nematode *B. xylophilus* [13,14,37].

In accordance with the strong toxicity to *M. incognita* and *P. vulnus* observed in our studies, EOs of *R. graveolens* and *S. aromaticum* were described as highly active on *J2* and eggs of *M. incognita*, *M. chitwoodi* Golden, O’Bannon, Santo and Finley, and *M. exigua* Goeldi [35,38,39], as well as on different stages of *B. xylophilus* [13,15]. Contrastingly, a greenhouse study of Meyer et al. [40] did not find any significant reduction of *M. incognita* population on various vegetable crops in soil treated with a *S. aromaticum* EO formulation.

The low activity of *M. piperita* EO in our experiments is also confirmed by literature data, which reported *a* poor in vitro toxicity of this EO to root-knot nematode *J2* and eggs and its scarce effectiveness on the infestation of *M. arenaria* Chitwood on tomato in soil [11,12,41].

The EOs from *P. asperum* and *R. officinalis* resulted highly toxic to *M. incognita* in all our in vitro and in vivo experiments, but were generally documented as poorly active on root-knot nematode *J2* and eggs by previous in vitro assays [10,11,12,33]. Contrastingly, experimental applications of *R. officinalis* EO to soil resulted in a strong reduction of the infestation of *M. incognita* on tomato [42] as well as of *M. javanica* and *P. brachyurus* (Godfrey) Filipjev and Schuurmans Stekhoven on soybean, *Glycine max* (L.) Merr. [43].

A consistent documentation is also available for the nematicidal properties of single EOs’ constituents. Carvacrol and its isomer thymol, as common components of EOs from several aromatic plants, were stated for numerous biological/pharmacological effects [44,45,46,47]. Data of Laquale et al. [26] proved a strong in vitro toxicity of carvacrol to infective stages of *M. incognita* and *P. vulnus* and its inhibitory effect on *M. incognita* egg hatch, confirming the strong in vitro toxicity of carvacrol to *M. incognita* and *M. javanica J2* and eggs described in literature studies [11,36,48]. In addition, *M. javanica* infestation on tomato were found to be strongly reduced or even completely suppressed by soil treatments with carvacrol [11,48]. Thymol was also described by a number of reports as highly toxic, though less than carvacrol, to infective stages of root-knot nematodes [25,26,36,49,50,51] and to the pine nematode *B. xylophilus* [52], while a low in vitro sensitivity to thymol was observed for *P. vulnus* [26]. In agreement, greenhouse studies on thymol application to soil described a sharp decline of population densities of *M. arenaria* and of the soybean cyst nematode *Heterodera glycines* Ichinohe [53].

Eugenol, the dominant (89.6%) constituent of *S. aromaticum* EO, is a 2-alkyl(oxy)phenol sharing with carvacrol and thymol either some chemical features and a strong nematotoxic effect. In vitro studies on *M. incognita* and *M. javanica* documented an almost complete *J2* mortality and a strong reduction of egg differentiation and hatch following treatments with eugenol, either alone or in synergistic combination with other EO’s constituents [37,51,54,55,56]. Consistently, treatments with eugenol in soil infested by *M. incognita* or *M. arenaria* were able to suppress female and egg density and gall formation on tomato roots [41,56]. In contrast, a low activity of eugenol was observed on other phytonematodes, such as *B. xylophilus* and *P. penetrans* Filipjev and Schuurmans Stekhoven [16,57].

The main constituent of *R. graveolens* EO, *2*-undecanone, was only stated for strong in vitro effects on root-knot nematode *J2* and eggs [57,58], while literature data on the nematicidal efficacy of *E*-cinnamaldehyde, the major component of *Cinnamomum* EOs, are referred to its strong in vitro activity on *B. xylophilus* [14] and a high suppressiveness to *M. incognita* infestation on soybean when applied to soil [59,60].

Nematicidal performance of the major components of *P. asperum* EO, i.e., linalool, citronellol, and geraniol, varied according to the tested nematode species, as they were proved for a strong in vitro and in vivo activity on *Meloidogyne* species [11,50,55,61], but only moderately active on the soil saprophytic nematode *Caenorhabditis elegans* Maupas and the lesion nematode *P. penetrans* [16]. A synergistic activity of these three compounds was also suggested by their lower activity on *M. incognita* compared with the whole *P. asperum* EO [10].

Contrasting data are available for 1,8-cineole and limonene, the main constituents of *E. globulus* and *C. sinensis* EOs, respectively, as well as for camphor and α-pinene. In our studies, 1,8-cineole was found highly toxic to *M. incognita*, *P. vulnus*, and *X. index* [25], while it resulted poorly active in other studies on root-knot nematode *J2* and eggs [36,49,50,51]. The poor activity of limonene on *M. incognita J2* detected in our experiments [25] was in agreement with the total inactivity on *M. javanica J2* reported by Santana et al. [34] but in contrast to the high toxicity of limonene to root-knot nematodes described by other reports [11,33,50]. Analogously, poor effects of camphor and α-pinene constantly observed by us on *M. incognita*, *P. vulnus*, and *X. index* [25] disagree with previous reports of a strong activity on the same root-knot species [49,50]. Regarding other EOs compounds not tested in our experiments, thujone isomers, i.e., the main constituents of the *A. herba-alba* EO, were indicated as moderately or poorly toxic to *M. incognita* and *M. javanica J2*, respectively, as well as not significantly active on other nematode species, such as *C*. *elegans* and *P*. *penetrans* [16,49]. The two main components of *M. piperita* EO, menthol and menthone, were also described for a limited in vitro toxicity to *M. incognita* and *M. javanica* [50,51,62]. Poor information was available on nematicidal activity of other EOs’ constituents, as limited to *M. chitwoodi* egg hatch inhibition by γ-terpinene and *o*-cymene [39] and to the in vitro fumigant activity of citronellal against *M. incognita* [63].

## 5. Structure-Activity Relationship: Some Considerations

Analysis of compositional profiles and nematicidal performances of the 16 EOs as well as of structure of their major constituents allowed some considerations on structure-activity relationships. As documented by specific studies, toxicity of natural terpenoids to nematode and bacterial and microbial systems is influenced by type and position of functional groups in their molecular structure [11,24,25,27,64]. In particular, it has been shown that biocidal activity of monoterpenoids is enhanced by the presence of an oxygen-related function (aldheyde, ketone, or alcohol group) in their molecule as well as by the presence of a double bond system which would favor biological processes involving transfer of electrons, thus increasing terpenes reactivity towards nematodes [27,64]. Consistently, our in vitro assays [25,27] evidenced that EOs rich in citronellal, citronellol, linalool, geraniol, and *E*-cinnamaldehyde, such as those from *C. verum*, *P. asperum,* and *E. citrodora,* were highly active against *M. incognita*. In agreement, the ketone monoterpenoid thujone was also strongly toxic to root-knot nematode *J2* and eggs in the in vitro experiments reported in literature [16,49]. Moreover, it has been described that acyclic monoterpenoids are more active than cyclic terpenoids with the same above functional groups [64]. This could explain the relatively lower nematicidal effect of menthol and menthane-type monoterpenoids or 1,8-cineole-containing EOs, such as those of *M. piperita* or *E. globulus* [24,27]. This was also consistent with the lower activity on *M. incognita J2* of *E. globulus* EO compared with *E. citriodora*, which was characterized by *E*-cinnamaldehyde. On the other hand, this was somehow in contrast with results of Avato et al. [25], which described a strong time-dependent toxicity of 1,8-cineole on the three nematodes *M. incognita, P. vulnus,* and *X. index.* In agreement with the above findings on structure-activity requirements, terpenes without reactive functional groups and EOs with a high content of them did not show a relevant nematicidal activity, as found in our studies for γ-terpinene, α-pinene, limonene, and *ο*-cymene as well as for the rich-in-limonene EOs of *C. aurantium* and *C. sinensis* [25,27].

Consistently with other studies [27,65], phenolics-containing EOs used in our investigations exerted a strong nematicidal activity, as observed for *S. aromaticum* EO, with a high content of the 2-alkyl(oxy) phenol eugenol, EOs of *M. didyma* and *M. fistulosa*, containing the phenolic monoterpene carvacrol, and for *T. satureioides* EO, which showed a high amount of thymol. Analogously, the toxicity of *T. satureioides* EO to *M. incognita* could be at least partially related to the presence of thymol. Reactivity of phenolic molecules is related with the redox properties given by the presence of the hydroxyl group in their aromatic ring and, possibly, by the presence of a spacing group, such as, for example, the methoxyl group in the eugenol structure. As demonstrated in a targeted study on its structure-activity relationship, the bioactivity of eugenol and related molecules is also dependent on the allylic double bond which, with the phenolic proton, is an essential structural feature for the molecule interaction with cellular systems [66]. Thus, the chemical structure of phenols plays an important role in their ability to scavenge free radicals and related reactive species formed in many physiological processes and the anti-oxidant activity exerted by these compounds can also reasonably be involved in their toxic effects to phytonematodes.

## 6. Conclusions

Analysis of literature studies and our experimental findings clearly indicate a high potential of EOs or their pure constituents as sources of new effective nematicidal products suitable for nematode management of high-value horticultural and fruit crops. These new EO-based nematicides could have promising market prospects, joining a high nematicidal performance to environmental safety related to a low toxicity on non-target vertebrates [67].

Despite these positive features, presence on the market of EOs-derived nematicides is still poor and is limited to a few products based on mixtures of synthetic analogues of EO’s components such as thymol, geraniol, and eugenol. Success of these potential products seems to be impeded by some unsolved key issues. Firstly, the poor knowledge of mechanisms of EOs activity and of nematode target sites. The hypotheses of an anticholinesterase activity or an alteration of cell membrane permeability suggested by literature data [11,68] have still not received confirmation from updated studies. A standardized quanti-qualitative composition of EOs raw materials, mainly achievable by using quality plant sources and appropriate extraction techniques, is also needed for ensuring homogeneity and reproducibility of nematicidal efficacy of derivative products [69]. Volatility and difficult vehiculation by irrigation water of EOs also makes the development of efficient stabilization processes necessary, such as EOs micro- or nanoencapsulation in biopolymers, to ensure slow release and water solubility of active constituents [70,71]. Finally, a simplification of the complex and expensive biopesticide registration procedures is strongly required, to encourage the development of potential EO-based nematicides by small or medium industrial companies, i.e., the most interested in biopesticide market niche.

## Figures and Tables

**Table 1:** LD_50_ values of tested essential oils at a 24 h exposure of *M. incognita J2*.

EOs	LD_50_ Values (μg mL^−1^)
*Artemisia herba-alba*	0.5
*Cinnamomum camphora*	22.9
*Cinnamomum verum*	0.1
*Citrus aurantium*	>> *
*Citrus sinensis*	>>
*Eucalyptus citriodora*	2.4
*Eucalyptus globulus*	26.7
*Mentha piperita*	20.7
*Monarda dydima*	1.0
*Monarda fistulosa*	1.0
*Pelargonium asperum*	13.0
*Rosmarinus officinalis*	51.8
*Ruta graveolens*	2.3
*Schinus molle*	22.6
*Syzygium aromaticum*	2.1
*Thymus saturejoides*	61.9

* >> = largely above the range of tested concentrations.

## Data Availability

The data presented in this study are available on request from the corresponding author. The data are not publicly available due to journals’ copyright.
